# Associations between Urban Sprawl and Life Expectancy in the United States

**DOI:** 10.3390/ijerph15050861

**Published:** 2018-04-26

**Authors:** Shima Hamidi, Reid Ewing, Zaria Tatalovich, James B. Grace, David Berrigan

**Affiliations:** 1 College of Architecture, Planning and Public Affairs, University of Texas at Arlington, Arlington, TX 76019, USA; 2 Department of City and Metropolitan Planning, University of Utah, Salt Lake City, UT 84112, USA; ewing@arch.utah.edu; 3 Division of Cancer Control and Population Sciences, Surveillance Research Program, National Cancer Institute, Bethesda, MD 20892, USA; tatalovichzp@mail.nih.gov; 4 Wetland and Aquatic Research Center, U.S. Geological Survey, Lafayette, LA 70506, USA; gracej@usgs.gov; 5 Division of Cancer Control and Population Sciences, Behavioral Research Program, National Cancer Institute, Bethesda, MD 20892, USA; berrigad@mail.nih.gov

**Keywords:** urban sprawl, compactness, mortality, built environment, life expectancy

## Abstract

In recent years, the United States has had a relatively poor performance with respect to life expectancy compared to the other developed nations. Urban sprawl is one of the potential causes of the high rate of mortality in the United States. This study investigated cross-sectional associations between sprawl and life expectancy for metropolitan counties in the United States in 2010. In this study, the measure of life expectancy in 2010 came from a recently released dataset of life expectancies by county. This study modeled average life expectancy with a structural equation model that included five mediators: annual vehicle miles traveled (VMT) per household, average body mass index, crime rate, and air quality index as mediators of sprawl, as well as percentage of smokers as a mediator of socioeconomic status. After controlling for sociodemographic characteristics, this study found that life expectancy was significantly higher in compact counties than in sprawling counties. Compactness affects mortality directly, but the causal mechanism is unclear. For example, it may be that sprawling areas have higher traffic speeds and longer emergency response times, lower quality and less accessible health care facilities, or less availability of healthy foods. Compactness affects mortality indirectly through vehicle miles traveled, which is a contributor to traffic fatalities, and through body mass index, which is a contributor to many chronic diseases. This study identified significant direct and indirect associations between urban sprawl and life expectancy. These findings support further research and practice aimed at identifying and implementing changes to urban planning designed to support health and healthy behaviors.

## 1. Introduction

Although the United States spends more per capita on health care than any other nation in the world, the life expectancy of its residents has fallen well below other developed countries [1]. In 1980, the United States was ranked 11th for life expectancy. However, by 1990 it had fallen to 13th, and by 2006 it was 21st [2]. In nearly half of U.S. counties, women today are not living as long as their mothers did [3]. Other research has shown that life expectancy is not changing uniformly across the United States. There are disparities of more than 20 years between counties with the highest and lowest life expectancies [4].

Several studies have sought to explain why life expectancy is shorter in the United States than in most other first-world nations [5,6,7,8,9,10,11,12,13,14,15,16,17,18,19]. These studies point to sociodemographic variables that affect life expectancy, including gender (women live longer than men) [8,9,10], income and socio-economic status (SES) [11] (people with higher incomes live longer than people with lower incomes) [12,13], race (whites live longer than non-whites) [14,15], and education (people with more education live longer than people with less) [16,17,18,19].

In addition to sociodemographic characteristics, specific behavioral and environmental modifiable risk factors such as smoking [2,20,21,22,23], obesity and associated chronic diseases [2,21], traffic fatalities [24,25], poor air quality [26,27,28,29], and homicide [30] contribute to reduced life expectancy. Smoking is the number one cause of preventable deaths [20] and both the number of cigarettes smoked and duration of smoking are strongly associated with all-cause mortality [23]. Obesity is the second leading preventable cause of death in the United States [21]. Traffic fatalities are the leading cause of death for those aged 15 to 24 years and are the sixth leading preventable cause of death in this country [24,25]. Many studies have related air quality to illness and premature death [26,27,28,29].

Most of these behavioral and environmental correlates of life expectancy are also associated with urban sprawl, a type of development pattern characterized by poor accessibility and automobile dependence. The United States has carried out a grand experiment by making most cities more sprawling over the past century. In the planning literature, urban sprawl is defined as the following urban forms: (1) leapfrog or scattered development; (2) commercial strip development; (3) expanses of low-density development; and (4) expanses of single-use development (as in bedroom communities, regional malls, and business parks). On the other hand, compact development is defined as a development pattern with strong centers, mixed land uses, medium-to-high densities, good accessibility, and permanent open spaces [31,32,33,34,35,36,37,38].

Obesity is higher in sprawling counties than in compact counties; several authors argue that reduced levels of physical activity and increased sedentary time could be responsible for the relationship between sprawl and obesity [31,32,33,34,35,36,37,38]. Traffic fatality rates are higher in sprawling than in compact counties due to increased exposure to driving and crashes [39]. At least four studies have related sprawl to poor air quality [40,41,42,43]. The most recent and most comprehensive study found that on average, metropolitan areas with lower levels of sprawl exhibit lower concentrations of ozone (O_3_) and fine particulates (PM_2.5_), which are known determinants of increased mortality [43]. Finally, violent crime rate may be lower in compact areas due to increased policing that tends to accompany population concentrations, along with factors such as community cohesion, availability of economic opportunities, and higher education and income levels of residents [44,45]. Jane Jacobs, an influential urban planner, has argued that dense neighborhoods produce “eyes on the street”, which in turn deters crime [46]. On the other hand, there is also evidence that homicide rates are higher in cities than in suburbs [47,48].

Although these observations suggest that urban sprawl may affect life expectancy, the literature provides little or no evidence on the exact nature of the relationship. Recently, the Institute of Health Metrics and Evaluation (IHME) at the University of Washington released a dataset of life expectancy at the county level for 1985–2010 [49]. Building on this dataset, this study seeks to test hypotheses about the connections between sprawl and life expectancy for metropolitan counties in the United States. The study uses structural equation modeling to estimate both direct and indirect associations between sprawl and life expectancy. If associations are established and verified by others, urban sprawl may emerge as another risk factor for premature death.

## 2. Materials and Methods

### 2.1. Data and Variables

The variables tested in the model are shown in Table 1. Descriptive statistics are computed, including percentages, means, and standard deviations for sociodemographic and built environmental variables. Our measure of life expectancy came from a recently released dataset of life expectancies by county [50].

Four mediating (endogenous) variables are posited between sprawl and life expectancy, and a fifth between socioeconomics and life expectancy. The first four are: average county-level vehicle miles traveled (VMT) per household [51], the U.S. Environmental Protection Agency’s (EPA) air quality index (AQI, a combination of six air quality indicators) [52], average county body mass index (BMI) [53], and the violent crime rate (crime) [54]. They relate, respectively, to four causes of premature death—traffic accidents, respiratory illnesses, obesity-related chronic health conditions, and crime and its effects on physical and mental health. The fifth mediating variable is the prevalence of smoking in the population, which has no obvious relationship to sprawl but a strong relationship to socioeconomic status.

County VMT estimates were obtained from the EPA. The EPA used surrogates such as population and roadway miles to allocate statewide total VMT to individual counties. Total VMT was divided by the number of households in each metropolitan county in 2010 to obtain VMT per household [51].

EPA has estimated AQI at the county level and includes an annual summary of days with good, moderate, unhealthy, and very unhealthy air. The AQI takes all six air pollutant criteria into account: carbon monoxide, nitrogen dioxide, ozone, sulfur dioxide, PM_2.5_, and PM_10_. The ratio of unhealthy days to total days is included as a variable in the model [52].

The BMI data came from the Behavioral Risk Factor Surveillance System (BRFSS), a telephone survey conducted by state health departments and managed by the Centers for Disease Control and Prevention (CDC). More than 350,000 adults are interviewed nationally each year to collect detailed information on health risk behaviors, preventive health practices, and health care access primarily related to chronic disease and injury. The Selected Metropolitan/Micropolitan Area Risk Trends (SMART) project, which is populated with BRFSS data for metropolitan and micropolitan statistical areas with 500 or more respondents was used in this study [53]. This study used the county average BMI estimated from data for survey years 2007 through 2010.

Crime statistics were obtained from the uniform crime report of the Federal Bureau of Investigation (FBI). The FBI supplies crime data by type (e.g., violent and property) and subtype (e.g., murder, rape, theft) aggregated by county. In this study, a violent crime rate is computed by dividing the total number of violent crimes by the county population in hundreds of thousands. The violent crime rate is treated as an endogenous variable [54].

Finally, smoking prevalence, the only endogenous variable unrelated to sprawl, has been estimated at the county level by the National Cancer Institute based on combined information from the two major health surveys, the BRFSS and the National Health Interview Survey (NHIS) [55]. The estimates are based on grouped years to provide reasonable sample sizes in each county. This study used the most recent time periods’ estimates, 2000–2003. The smoking prevalence variable in this study is “ever smoked”. For ever smoked, a person 18 years of age or older must have reported smoking at least 100 cigarettes in their lifetime by the time of interview, in both BRFSS and NHIS surveys [55]. This study treats smoking as a mediating (endogenous) variable on the pathway between sociodemographic variables and life expectancy.

Exogenous variables came from various sources. From the 2010 Census, we downloaded data on population, households, sex, age, and race/ethnicity, and computed percentage of the population that is male and percentage of the population that is white. In this study, the Yost et al. SES index was modified as a measure of socioeconomic status [56,57]. Yost included SES as a composite factor that combines three generally accepted domains: education, income, and occupation. For this study, we updated Yost’s index using census 2010 data.

The exogenous variable of greatest interest is the county compactness/sprawl index. This index places urban sprawl at one end of a continuous scale and compact development at the other [59]. The original index, developed in 2002, was updated to 2010 in a recent study [58,60,61]. The updated index incorporates more measures of the built environment than the original index did, and captures four distinct dimensions of sprawl: development density; land use mix; population and employment centering; and street accessibility, which represents the relative connectivity of the street network at the county level. These four dimensions are extracted from multiple correlated variables using principal component analysis and the first principal component is transformed to an index with the mean of 100 and a standard deviation of 25. The National Institutes of Health website [62] provides detailed information on the methodology, variable names under each dimension, factor loadings (the correlation between a variable and a principal component), eigenvalues (the explanatory power of a single principal component), and percentages of explained variance. This updated index is freely available for 994 counties and county equivalents [62]. The updated index was used as the measure of compactness in this study.

### 2.2. Statistical Analysis

This study used structural equation modeling (SEM) to address associations between life expectancy and urban sprawl. SEM is a “model-centered” methodology that seeks to evaluate theoretically-justified models against data [63,64]. The estimation of SEM models involves solving a set of equations, one for each “response” or “endogenous” variable in the network. Variables that are solely predictors of other variables are termed “influences” or “exogenous” variables.

A SEM model for life expectancy was estimated using Amos 19 and maximum likelihood procedures. A total of 606 metropolitan counties with no missing data were included in the analysis. Working with complete datasets allowed us to compute modification indices, which in turn allowed us to identify missing links in the model. Modification indices are computable only if the dataset contains no missing information. Data were examined for frequency distributions and simple bivariate relationships, especially for linearity. All variables were natural log (ln) transformed to equalize variances and improve linearity.

Four plausible mediating pathways were included connecting sprawl with life expectancy. One pathway was through average county VMT, used as a proxy for traffic fatalities in the SEM model. A second pathway was through air pollution. Air Quality Index was used as the measure of air pollution. A third pathway was through obesity, which was measured as the average BMI. A fourth and final pathway was through the violent crime rate.

This study reports the following measures of fit: the chi square, the root mean square error of approximation (RMSEA), and the comparative fit index (CFI). This study also reports results as standardized regression coefficients, which represent a standard deviation of change in the outcome per standard deviation of change in the independent variable.

## 3. Results

**Figure 1:**
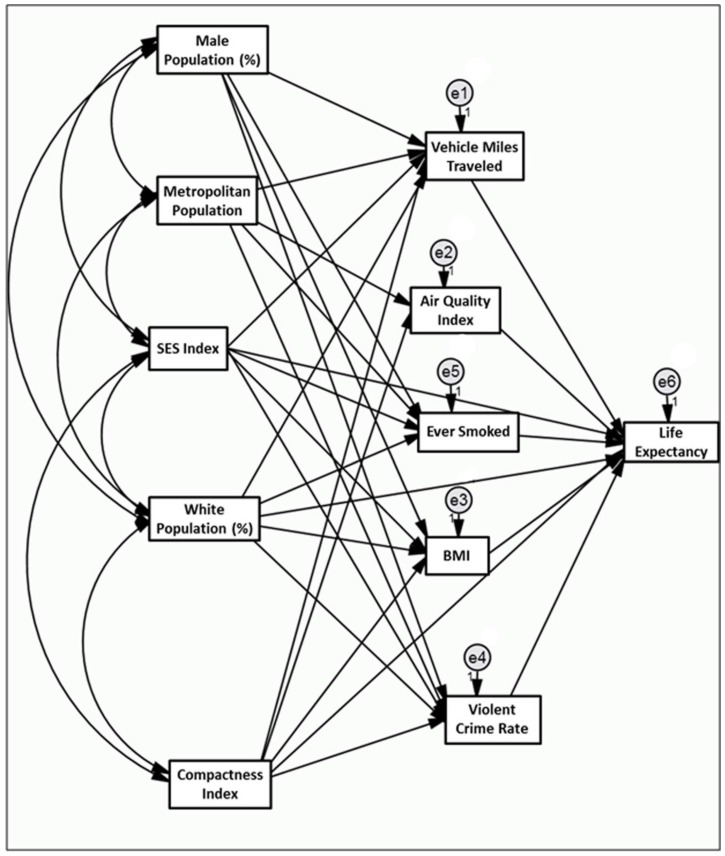
Associations between county level sprawl and life expectancy incorporating VMT, air quality index (AQI), average county body mass index (BMI), smoking, and violent crime as mediators.

The best fitted model is shown in Figure 1. Directional pathways are represented by straight uni-directional arrows. Correlations are represented by curved bi-directional arrows (to simplify the already complex diagram, some correlations are omitted from the diagram but not the model as presented in Table 2). By convention, circles represent error terms in the model, of which there is one for each endogenous (response) variable.

Judged by goodness-of-fit measures (significant coefficients, low model chi-square, and sample-size adjusted fit (the RMSEA)), the model fit the data well. The model had a low chi-square of 18.6 with 13 model degrees of freedom and a high *p*-value of 0.136. The low chi-square relative to model degrees of freedom and a high (>0.05) *p*-value are indicators of good model fit. The CFI value shows that the model explains most of the total discrepancy in the data (>93%).

The regression coefficients for the sociodemographic and built environmental variables are provided in Table 2. Most of the relationships are highly significant and generally meet expectations. The county compactness index had a direct positive association with life expectancy, plus indirect effects through mediating variables. Compactness was directly associated with mortality, but the potential mechanisms are unclear. We speculate about the reasons for this significant relationship in the Discussion section. The compactness index was inversely related to VMT because origins and destinations are closer together in a compact county. In turn, VMT was negatively related to life expectancy, which means that the indirect effect of compactness on life expectancy through this compound pathway was positive. The compactness index was also negatively related to BMI, which was inversely related to life expectancy, meaning that through this pathway, compactness had a positive indirect effect on life expectancy. The compactness index was positively related to the AQI, which was negatively related to life expectancy. This means that air quality was worse in compact counties and life expectancies may be shorter. However, the latter relationship was not statistically significant, so the indirect effect of compactness on life expectancy through this pathway was negative but weak. The compactness index was positively related to violent crime, which was negatively related to life expectancy, meaning that the indirect effect of compactness on life expectancy through this pathway was also negative. Finally, the rate of smoking had a direct negative effect on life expectancy, with no connection to compactness.

The direct, indirect, and total effects of the county compactness index and other variables on life expectancy are shown in Table 3. The net indirect effect of compactness on life expectancy was positive, and even more positive when added to the direct effect. We conclude that life expectancy was significantly higher in compact than in sprawling counties. A life expectancy increase of three percent was seen with a doubling of the compactness index (increase by 100%). For the average American with a life expectancy of 78 years, this translates into a two-and-half year difference. That is well within the range of life expectancy differences from county to county.

## 4. Discussion

Investigators have long been interested in urban–rural differences in health and life expectancy. This work examining associations between life expectancy and aspects of the urban environment is a logical next step. Approximately half of the world’s population currently lives in cities, and this value is expected to continue to climb. Projections by the United Nations suggest that 60 percent of the world’s population will reside in urban regions by 2030. High rates of urbanization could mean increasing numbers of people affected by urban sprawl if steps are not taken to contain it.

This study is the first attempt to understand the relationship between urban sprawl and life expectancy, taking both direct and indirect effects into account. The major question, which we cannot answer, is why sprawl directly affects life expectancy beyond the indirect effects this study has already measured. There could be additional indirect effects due to unmeasured covariates. For example, it may be that sprawling areas generate higher traffic speeds and longer emergency response times, and this affects fatal traffic accidents. A measure of VMT alone does not capture this possibility. It may be that health services are better and more accessible in compact areas, because compact areas provide larger potential patient pools. It may be that healthy food is more available in compact areas, again due to market forces.

This study also found that life expectancy was significantly higher in compact than sprawling counties due to indirect effects, specifically due to lower VMT and BMI in compact counties. These associations are to be expected based on prior literature, which indicates that sprawl increases VMT [58], which is related to fatal traffic accidents [65], and BMI [31,32,33,34,35,36,37,38], which is related to chronic diseases such as coronary heart disease [2]. These results also are consistent with findings from previous studies showing that life expectancy is reduced by violent crime [30]. However, this study found that this indirect effect operates in the opposite direction of the earlier two. Violent crime rates were lower in sprawling counties and this increased life expectancy. There may also be a relationship between sprawl and air quality that favors sprawling counties in terms of life expectancy. Nonetheless, the effects of crime and air quality were small compared to those of VMT and BMI, and the indirect effect of sprawl on life expectancy was small compared to the direct effect.

A review of the large body of literature on geographic variation in life expectancy in the United States (e.g., [2,7,11]) is beyond the scope of this paper, but these results suggest that urban form may play a role in such variation. Results of these past studies linking health care, health behaviors, and life expectancy have been mixed. Recent reviews and analyses of county-level disparities in mortality have highlighted the role of tobacco use in women [2,3], specific chronic diseases and injuries [4], and urban–rural disparities [66]. On the other hand, the role of insurance status highlights the challenges of ecological analyses. Berrigan et al. (2014) report that insurance coverage is negatively correlated with cancer mortality [67], confirming the results of other ecological studies, but the opposite of extensive evidence at the individual level [68]. Multi-level analyses and examination of regional variation in sprawl and life expectancy could help clarify the interaction between individual and environmental factors as influences on longevity.

This study is subject to several limitations. First, progress in identifying modifiable factors associated with life expectancy requires stronger evidence for causal associations. This paper presents the results of a cross-sectional analysis of a novel potential determinant of geographic variation in life expectancy related to urban form. Second, this paper does not attempt to develop a complete model of factors associated with geographic variation in life expectancy. The mediating variables examined here are only four of many predictors of life expectancy. Key missing variables include variations in diet, physical activity, and access to health care. Past results concerning the association between mortality and health care at the county level have been mixed. This study also does not account for street-scale urban design variables such as sidewalks and topography that may act directly or interact to influence physical activity and hence obesity and life expectancy. Nor did this study examine regional variation in the potential association between sprawl and life expectancy. Future studies could examine such variation, as there appears to be regional variation in the association between sprawl and obesity-related cancer mortality in the United States.

## 5. Conclusions

Despite very high levels of expenditure on health care, the U.S is not among the countries with the highest life expectancies. Changes in urban form are a potential intervention to address health in urban areas. In the words of a recent editorial in the American Journal of Public Health, “Progressive trends in architecture and urban planning, including the green building movement, smart growth, and new urbanism, grew out of environmental and social goals but often promoted healthy design (sometimes incidentally, sometimes by intent) [69]”. If the results of this study and other related work are confirmed, there will be further reasons to promote alternatives to sprawling patterns of urban development “by intent” rather than incidentally.

## Figures and Tables

**Table 1:** Variables used to explain life expectancy (variables log transformed).

Variable	Abbreviation	Data Sources	Mean (SD)
Endogenous Variables			
Average life expectancy	Life expectancy	IMHE 2010 [50]	78.14 (2.03)
Annual vehicle miles traveled per household	VMT per household	EPA 2011 [51]	27,015 (8500)
Air quality index	Air quality index	EPA 2010 [52]	1.72 (3.48)
Average body mass index	Body mass index	BRFSS 2010 [53]	30.99 (2.01)
Violent crime rate per 100,000 population	Violent crime rate	FBI Uniform Crime Statistics 2010 [54]	346.17 (230.6)
Ever smoked	Ever smoked	NIH 2003 [55]	0.471 (0.057)
Exogenous Variables			
Metropolitan population	Metropolitan Pop.	Census 2010	1,931,779 (3,340,260)
Socio-economic status (SES) index	SES index	Yost et al. (2001) [56,57]	37,480 (7936)
Percentage of white population	White Pop. (%)	Census 2010	78.25 (14.94)
Percentage of male population	Male Pop. (%)	Census 2010	49.17 (1.07)
County compactness index for 2010	Compactness index	Ewing and Hamidi, 2017 [58]	106.76 (19.84)

**Table 2:** Direct effects of variables on one another in the life expectancy model.

**Variables**			**Estimate (Standard Error)**	**Critical Ratio**	***p*** **-Value**
**Demographic Variables**					
Metropolitan Pop.	→	Air quality index	0.13 (0.02)	6.613	<0.001
Metropolitan Pop.	→	VMT per household	−0.013 (0.008)	−1.597	0.110
Metropolitan Pop.	→	Violent crime rate	−0.052 (0.02)	−2.65	0.008
Metropolitan Pop.	→	Ever smoked	−0.01 (0.004)	−2.695	0.007
White Pop. (%)	→	Body mass index	−0.02 (0.012)	−1.563	0.118
White Pop. (%)	→	Ever smoked	0.262 (0.021)	12.179	<0.001
White Pop. (%)	→	VMT per household	−0.295 (0.049)	−6.017	<0.001
White Pop. (%)	→	Violent crime rate	−1.338 (0.116)	−11.571	<0.001
White Pop. (%)	→	Life expectancy	0.029 (0.004)	7.683	<0.001
Male Pop. (%)	→	VMT per household	0.472 (0.475)	0.994	0.320
Male Pop. (%)	→	Ever smoked	−0.847 (0.219)	−3.875	<0.001
Male Pop. (%)	→	Body mass index	−0.019 (0.119)	−0.163	0.871
Male Pop. (%)	→	Violent crime rate	−1.448 (1.119)	−1.294	0.196
SES index	→	VMT per household	0.121 (0.048)	2.534	0.011
SES index	→	Violent crime rate	−0.634 (0.113)	−5.604	<0.001
SES index	→	Ever smoked	−0.126 (0.022)	−5.663	<0.001
SES index	→	Body mass index	−0.063 (0.01)	−6.005	<0.001
SES index	→	Life expectancy	0.05 (0.003)	16.805	<0.001
**Compactness Index**					
Compactness index	→	VMT per household	−0.956 (0.059)	−16.344	<0.001
Compactness index	→	Air quality index	0.553 (0.15)	3.682	<0.001
Compactness index	→	Body mass index	−0.042 (0.015)	−2.866	<0.001
Compactness index	→	Violent crime rate	1.162 (0.138)	8.441	<0.001
**Risk Factors**					
VMT per household	→	Life expectancy	−0.015 (0.003)	−5.443	<0.001
Air quality index	→	Life expectancy	−0.001 (0.001)	−1.293	0.196
Ever smoked	→	Life expectancy	−0.08 (0.006)	−14.073	<0.001
Body mass index	→	Life expectancy	−0.024 (0.011)	−2.281	0.023
Violent crime rate	→	Life expectancy	−0.006 (0.001)	−5.236	<0.001
Compactness index	→	Life expectancy	0.022 (0.005)	4.587	<0.001

Analysis was restricted to counties without missing values. Chi-square = 18.6; degrees of freedom = 13; *p*-value = 0.136; root mean square error of approximation (RMSEA) = 0 (*p*-value = 0.933); comparative fit index (CFI) = 0.997.

**Table 3:** Direct, indirect, and total effects of the county compactness index and other variables on life expectancy.

Variable	Direct Effect	Indirect Effect	Total Effect
Metropolitan Pop	0	0.001	0.001
White Pop (%)	0.029	−0.008	0.021
Male Pop (%)	0.010	0.070	0.080
SES index	0.051	0.014	0.064
VMT per household	−0.015	0	−0.015
Air quality index	−0.001	0	−0.001
Ever smoked	−0.080	0	−0.080
Body mass index	−0.024	0	−0.024
Violent crime rate	−0.006	0	−0.006
Compactness index	0.022	0.007	0.030

## References

[1] World Health Organization (2013). World Health Statistic Annual 2013.

[2] Crimmins E.M., Preston S.H., Cohen B. (2011). Explaining Divergent Levels of Longevity in High-Income Countries.

[3] Kindig D.A., Cheng E.R. (2013). Even as mortality fell in most US counties, female mortality nonetheless rose in 42.8 percent of counties from 1992 to 2006. Health Aff..

[4] Murray C.J., Kulkarni S.C., Michaud C., Tomijima N., Bulzacchelli M.T., Iandiorio T.J., Ezzati M. (2006). Eight Americas: Investigating mortality disparities across races, counties, and race-counties in the United States. PLoS Med..

[5] Mirowsky J., Ross C. (2000). Socioeconomic Status and Subjective Life Expectancy. Soc. Psychol. Q..

[6] Ezzati M., Friedman A., Kulkarni S., Murray C. (2008). The Reversal of Fortunes: Trends in County Mortality and Cross-County Mortality Disparities in the United States. PLoS Med..

[7] Olshansky S.J., Antonucci T., Berkman L., Binstock R.H., Boersch-Supan A., Cacioppo J.T., Carnes B.A., Carstensen L.L., Fried L.P., Goldman D.P. (2012). Differences in life expectancy due to race and educational differences are widening, and many may not catch up. Health Aff..

[8] Crimmins E.M., Saito Y. (2001). Trends in healthy life expectancy in the United States, 1970–1990: Gender, racial, and educational differences. Soc. Sci. Med..

[9] Olshansky S.J., Carnes B.A., Désesquelles A. (2001). Demography: Prospects for human longevity. Science.

[10] Olshansky S.J., Passaro D.J., Hershow R.C., Layden J., Carnes B.A., Brody J., Hayflick L., Butler R.N., Allison D.B., Ludwig D.S. (2005). A potential decline in life expectancy in the United States in the 21st century. N. Engl. J. Med..

[11] Swanson D.A., Sanford A.G. (2012). Socio-Economic Status and Life Expectancy in the United States, 1990–2010: Are We Reaching the Limits of Human Longevity?. Popul. Rev..

[12] Mellor J.M., Milyo J. (2002). Income inequality and health status in the United States: Evidence from the current population survey. J. Hum. Resour..

[13] Wilkinson R.G., Pickett K.E. (2006). Income inequality and population health: A review and explanation of the evidence. Soc. Sci. Med..

[14] Harper S., Lynch J., Burris S., Smith G.D. (2007). Trends in the black-white life expectancy gap in the United States, 1983–2003. JAMA.

[15] Danaei G., Rimm E., Oza S., Kulkarni S., Murray C., Ezzati M. (2010). The promise of prevention: The effects of four preventable risk factors on national life expectancy and life expectancy disparities by race and county in the United States. PLoS Med..

[16] Christenson B., Johnson N. (1995). Educational Inequality in Adult Mortality: An Assessment with Death Certificate Data from Michigan. Demography.

[17] Elo I., Preston S. (1996). Educational Differentials in Morality: United States, 1979–85. Soc. Sci. Med..

[18] Manton K.G., Stallard E., Martin L.G., Soldo B.J. (1997). Health and disability differences among racial and ethnic groups. National Research Council, Racial and Ethnic Differences in the Health of Older Americans.

[19] Meara E.R., Richards S., Cutler D.M. (2008). The gap gets bigger: Changes in mortality and life expectancy, by education, 1981–2000. Health Aff..

[20] US Department of Health and Human Services (2004). The Health Consequences of Smoking: A Report of the Surgeon General.

[21] Stewart S.T., Cutler D.M., Rosen A.B. (2009). Forecasting the effects of obesity and smoking on US life expectancy. N. Engl. J. Med..

[22] Kaplan R.M., Anderson J.P., Kaplan C.M. (2007). Modeling quality-adjusted life expectancy loss resulting from tobacco use in the United States. Soc. Indic. Res..

[23] Streppel M.T., Boshuizen H.C., Ocké M.C., Kok F.J., Kromhout D. (2007). Mortality and life expectancy in relation to long-term cigarette, cigar and pipe smoking: The Zutphen Study. Tob. Control.

[24] Mokdad A.H., Marks J.S., Stroup D.F., Gerberding J.L. (2004). Actual causes of death in the United States, 2000. JAMA.

[25] Kochanek K.D., Xu J., Murphy S.L., Miniño A.M., Kung H.C. (2011). National vital statistics reports. Natl. Vital Stat. Rep..

[26] Levy J.I., Carrothers T.J., Tuomisto J.T., Hammitt J.K., Evans J.S. (2001). Assessing the public health benefits of reduced ozone concentrations. Environ. Health Perspect..

[27] World Health Organization (2003). Health Aspects of Air Pollution with Particulate Matter, Ozone and Nitrogen Dioxide.

[28] Dockery D.W. (2009). Health effects of particulate air pollution. Ann. Epidemiol..

[29] Silva R.A., West J.J., Zhang Y., Anenberg S.C., Lamarque J.F., Shindell D.T., Collins W.J., Dalsoren S., Faluvegi G., Folberth G. (2013). Global premature mortality due to anthropogenic outdoor air pollution and the contribution of past climate change. Environ. Res. Lett..

[30] Redelings M., Lieb L., Sorvillo F. (2010). Years off your life? The effects of homicide on life expectancy by neighborhood and race/ethnicity in Los Angeles County. J. Urban Health.

[31] Ewing R., Schmid T., Killingsworth R., Zlot A., Raudenbush S. (2003). Relationship between Urban Sprawl and Physical Activity, Obesity, and Morbidity. Am. J. Health Promot..

[32] Lopez R. (2004). Urban sprawl and risk for being overweight or obese. Am. J. Public Health.

[33] Sturm R., Cohen D.A. (2004). Suburban sprawl and physical and mental health. Public Health.

[34] Alley D.E., Lloyd J., Shardell M., Crimmins E.M., Preston S.H., Cohen B. (2010). Can obesity account for cross-national differences in life expectancy trends?. National Research Council, International Differences in Mortality at Older Ages: Dimensions and Sources.

[35] Feng J., Glass T.A., Curriero F.C., Stewart W.F., Schwartz B.S. (2010). The built environment and obesity: A systematic review of the epidemiologic evidence. Health Place.

[36] Papas M.A., Alberg A.J., Ewing R., Helzlsouer K.J., Gary T.L., Klassen A.C. (2007). The built environment and obesity. Epidemiol. Rev..

[37] Black J.L., Macinko J. (2008). Neighborhoods and obesity. Nutr. Rev..

[38] Ewing R., Meakins G., Hamidi S., Nelson A.C. (2014). Relationship between urban sprawl and physical activity, obesity, and morbidity—Update and refinement. Health Place.

[39] Ewing R., Schieber R., Zegeer C. (2003). Urban Sprawl as a Risk Factor in Motor Vehicle Occupant and Pedestrian Fatalities. Am. J. Public Health.

[40] Stone B. (2008). Urban Sprawl and Air Quality in Large U.S. Cities. J. Environ. Manag..

[41] Ewing R., Pendall R., Chen D. (2002). Measuring Sprawl and Its Impacts.

[42] Schweitzer L., Zhou J. (2010). Neighborhood Air Quality Outcomes in Compact and Sprawled Regions. JAPA.

[43] Bereitschaft B., Debbage K. (2013). Urban Form, Air Pollution, and CO_2_ Emissions in Large U.S. Metropolitan Areas. Prof. Geogr..

[44] Browning C.R., Byron R.A., Calder C.A., Krivo L.J., Kwan M.P., Lee J.Y., Peterson R.D. (2010). Commercial density, residential concentration, and crime: Land use patterns and violence in neighborhood context. J. Res. Crime Delinq..

[45] Litman T. Safer than you think! Revising the transit safety narrative. Presented at the 92th Annual Meeting of the Transportation Research Board.

[46] Jacobs J. (1961). The Death and Life of Great American Cities.

[47] Lucy W.H. (2003). Mortality Risk Associated with Leaving Home: Recognizing the Relevance of the Built Environment. Am. J. Public Health.

[48] Myers S.R., Branas C.C., French B.C., Nance M.L., Kallan M.J., Wiebe D.J., Carr B.G. (2013). Safety in numbers: Are major cities the safest places in the United States?. Ann. Emerg. Med..

[49] Wang H., Schumacher A.E., Levitz C.E., Mokdad A.H., Murray C.J. (2013). Left behind: Widening disparities for males and females in US county life expectancy, 1985–2010. Popul. Health Metr..

[50] Institute for Health Metrics and Evaluations, County Level Life Expectancy Database. http://www.healthmetricsandevaluation.org/publications/summaries/left-behind-widening-disparities-males-and-females-us-county-life-expectancy-#/data-methods.

[51] Environmental Protection Agency’s VMT Estimates. http://www.epa.gov/pmdesignations/2012standards/docs/vmt2011.xlsx.

[52] Environmental Protection Agency’s Air Quality Index. https://www.epa.gov/outdoor-air-quality-data.

[53] Behavioral Risk Factor Surveillance System (BRFSS) Survey. http://www.cdc.gov/brfss/smart/smart_data.htm.

[54] Federal Bureau of Investigation (FBI) Crime Statistics. http://www.ucrdatatool.gov/.

[55] Small Area Estimates for Cancer-Related Measures. http://sae.cancer.gov/estimates/lifetime.html.

[56] Yost K., Perkins C., Cohen R., Morris C., Wright W. (2001). Socioeconomic status and breast cancer incidence in California for different race/ethnic groups. Cancer Causes Control.

[57] Yu M., Tatalovich Z., Gibson J.T., Cronin K.A. (2014). Using a composite index of socioeconomic status to investigate health disparities while protecting the confidentiality of cancer registry data. Cancer Causes Control.

[58] Ewing R., Hamidi S. (2017). Costs of Sprawl.

[59] Ewing R., Pendall R., Chen D. (2003). Measuring sprawl and its transportation impacts. Transp. Res. Rec..

[60] Ewing R., Hamidi S. (2014). Measuring Urban Sprawl and Validating Sprawl Measures.

[61] Ewing R., Hamidi S., Grace J.B. (2016). Urban sprawl as a risk factor in motor vehicle crashes. Urban Stud..

[62] Ewing and Hamidi Compactness Index. http://gis.cancer.gov/tools/urban-sprawl.

[63] Grace J.B. (2006). Structural Equation Modeling and Natural Systems.

[64] Hoyle R.H. (2012). Handbook of Structural Equation Modeling.

[65] Ewing R., Dumbaugh E. (2009). The Built Environment and Traffic Safety: A Review of Empirical Evidence. J. Plan. Lit..

[66] Singh G.K., Siahpush M. (2014). Widening Rural–Urban Disparities in Life Expectancy, US, 1969–2009. Am. J. Prev. Med..

[67] Berrigan D., Tatalovich Z., Pickle L.W., Ewing R., Ballard-Barbash R. (2014). Urban sprawl, obesity, and cancer mortality in the United States: Cross-sectional analysis and methodological challenges. Int. J. Health Geogr..

[68] Marlow N.M., Pavluck A.L., Bian J., Ward E.M., Halpern M.T. (2009). The Relationship between Insurance Coverage and Cancer Care: A Literature Synthesis.

[69] Jackson R.J., Dannenberg A.L., Frumkin H. (2013). Health and the Built Environment: 10 Years After. Am. J. Public Health.

